# Loneliness in Myanmar’s older population: A mixed-methods investigation

**DOI:** 10.1007/s10823-022-09459-x

**Published:** 2022-10-27

**Authors:** Samia C. Akhter-Khan, Khin Myo Wai, Johanna Drewelies

**Affiliations:** 1 Department of Health Service & Population Research, King’s College London, London, UK; 2 Faw Htoo Kaw Consultancy, Yangon, Myanmar; 3 Department of Psychology, Humboldt University of Berlin, Berlin, Germany; 4 Lise Meitner Group for Environmental Neuroscience, Max-Planck-Institute for Human Development, Berlin, Germany

**Keywords:** Ageism, Healthy Aging, Global Mental Health, Social Relationships, Southeast Asia

## Abstract

**Objectives:**

Little is known about loneliness in lower- and middle-income countries. This study investigates loneliness in the older population of Myanmar using a mixed-methods approach.

**Methods:**

To identify predictors of loneliness, hierarchical regression models were used to analyze data from the Myanmar Aging Survey 2012 (N = 3,618, 57% women). In a mixed-methods sequential explanatory design, quantitative data were integrated with qualitative data from semi-structured interviews with older adults in Myanmar in 2019.

**Results:**

The prevalence of loneliness varied by between-person characteristics. Health impairments, lower income, being widowed, not having children, and living with fewer household members were each associated with loneliness. Qualitative findings suggested that the physical presence of family members was especially protective against loneliness. Religion had mixed associations with loneliness, depending on the type of religious practice, demographic characteristics, health status, and community engagement.

**Discussion:**

The findings contribute to a better understanding of individuals’ experiences of loneliness and may inform the design of interventions to prevent loneliness in Myanmar and globally.

**Supplementary Information:**

The online version contains supplementary material available at 10.1007/s10823-022-09459-x.

## Introduction

In recent years, loneliness has become of increasing interest to researchers and public policymakers due to its strong associations with detrimental health outcomes (e.g., Holt-Lunstad et al., 2015). Loneliness is defined as the subjective feeling resulting from the perceived discrepancy between one’s desired versus actual social relationships (Peplau & Perlman, 1982). Importantly, loneliness is distinct from social isolation, which only refers to the *objective* state of being alone, not the *subjective* appraisal of this state. This distinction is important, as loneliness is more strongly linked to negative health outcomes, such as dementia and cardiovascular disease (CVD), than social isolation (Bu et al., 2020; Rafnsson et al., 2020). Understanding the risk factors and mechanisms of loneliness will strengthen efforts to prevent illness and promote healthy aging.

To date, however, efforts to prevent and reduce loneliness in old age with targeted interventions have not been very effective (Akhter-Khan & Au, 2020; Masi et al., 2011). The limitation of these efforts was that they did not give sufficient attention to contextual factors surrounding loneliness, which, as recent research has shown, impact the experience of loneliness in old age (e.g., Masi et al., 2011). For one, older people are more likely than younger people to experience adverse life events, such as bereavement and impaired health (e.g., Mund et al., 2020). Indeed, widowhood and impaired health were among the most robust predictors of loneliness in a meta-analysis (Cohen-Mansfield, Hazan, Lerman, & Shalom, et al., 2016). In addition, loneliness is linked to a variety of factors that tend to increase in old age, such as lower income, reduced quantity of social relationships, and residence in rural areas (Cohen-Mansfield et al., 2016).

However, the aforementioned studies were themselves limited by the fact that they focused only on subjects from WEIRD (Western, Educated, Industrialized, Rich, and Democratic) countries (Henrich et al., 2010). The lack of knowledge about loneliness in non-WEIRD countries is troubling given that globally, and especially in lower- and middle-income countries (LMICs), such as Myanmar, population aging is on the rise and posing tremendous threats to older people’s well-being (Beller & Wagner, 2020; United Nations, 2015). In addition, even the work on loneliness that has been conducted in non-WEIRD countries may still not be generalizable to Myanmar, a culturally diverse nation that only recently opened up to the international community—a move that has led to rapid social, economic, and political changes. In order to design effective interventions for loneliness in Myanmar—or any other country—it will be necessary to consider the contextual factors specific to the situation (Haroz et al., 2017). Below, we describe contextual and cultural factors specific to Myanmar as well as the hypotheses about predictors of loneliness that we generated based on these factors.

### Country Context

Decades of political turmoil, conflicts, and inequalities have led Myanmar to become one of the poorest and least healthy countries in Southeast Asia (Nguyen et al., 2018; Teerawichitchainan & Knodel, 2015). Older people’s mental health has been a largely neglected topic, and interventions are sparse (Nguyen et al., 2018; Yamada et al., 2020). With limited access to long-term care (LTC), older adults in Myanmar rely on their communities for securing their physical and mental health. Approximately 86% of older people live in multi-generational arrangements (Knodel & Nguyen, 2015); for these adults, intergenerational support structures likely play a large role in ensuring their well-being and preventing loneliness. Indeed, family interactions and loneliness are more strongly linked in collectivistic societies than in individualistic societies (Beller & Wagner, 2020).

#### The Role of Family Network, Religion, and Sociodemographic Characteristics for Loneliness

**Family***.* One example of a cultural practice unique to Myanmar is the tendency for unmarried older adults to live with a sibling’s family, not alone. This cultural norm is reflected in Burmese (Myanmar’s most widely spoken language) itself: Unmarried older adults are affectionately called “older singles” (a pyo kyi for women, lu pyo kyi for men). Indeed, older people without their own children still described themselves as “parents” (e.g., as parents of their siblings’ children, adopted, or stepchildren) in the most recent Population and Housing Census from 2014 (Department of Population, 2017). This fact may reflect the disassociation between marital status and household composition in Myanmar culture, as older people without partners or spouses often live with other family members and take on roles as alloparents (i.e., any group member other than the biological parents who participates in the upbringing of children; Hrdy 2009). As such, in our study, we predicted that household composition would be a stronger predictor of loneliness than marital status or having children.

**Religion**. Religion plays a major role in older people’s well-being in Myanmar. Almost 90% of Myanmar’s population are Theravada Buddhists who regularly practice meditation, a practice shown to promote mental health (Nguyen et al., 2018; Schödwell et al., 2019). Other religious groups, such as Christians, Muslims, and Hindus, are also prevalent in Myanmar’s society and likely also turn to religious practices and organizations for support with mental health issues (Nguyen et al., 2018). Furthermore, religious concepts are known to shape the cultural understanding of depression in Myanmar (Schödwell et al., 2019); plausibly, the same may be true for loneliness, although little research has explored whether this is the case. A recent report also showed that there exists a cultural expectation for older people in Myanmar to participate in religious activities as a form of community engagement (HelpAge International, 2019), which suggests that older people may potentially feel lonely when they cannot meet this societal expectation—due to health impairments, for instance.

**Gender Differences***.* Another cultural factor in Myanmar is the presence of strong gender differences in widowhood status, household composition, and financial situation (Department of Population, 2017). According to the 2014 Census, less than half of older women were married, compared to 73.9% of same-aged men—a gap that is partially explained by differences in life-expectancy. For older women, widowhood becomes increasingly likely with age; among the 85–89-year-olds, 74% were widowed. With older women at particular risk to be bereaved, and widowhood as one of the strongest predictors of loneliness, we expected to find a direct effect of widowhood on loneliness as well as a gender mediation on other main effects (e.g., economic status, social activities).

**Socio-demographic Variables**. Socio-demographic variables related to population aging, ageism, and declining support ratios may increase older people’s risk of feeling lonely. Although only 7% of older people in Myanmar lived alone in 2012, the numbers are expected to sharply increase in the coming years due to demographic changes, as seen in neighboring Thailand (Knodel & Pothisiri, 2014). For one, younger people are increasingly migrating to urban areas or neighboring countries for employment, leaving their parents behind (Teerawichitchainan & Knodel, 2018). Although a recent cross-sectional study from Myanmar did not find differences in general psychological well-being among grandparents in skipped-generation households, compared to other grandparents (Teerawichitchainan & Low, 2021), urban migration is likely to have an effect on older adults’ loneliness. Moreover, ageism, i.e., negative attitudes and treatment of older people in employment, healthcare, financial, and societal sectors, seems to be on the rise in Myanmar (HelpAge International, 2019) and may also contribute to loneliness (Shiovitz-Ezra et al., 2018). Furthermore, older people who live alone or in poverty are particularly vulnerable to adverse health outcomes, which are known to be linked to feelings loneliness (Akhter-Khan & Wai, 2020; Knodel & Teerawichitchainan 2017; Teerawichitchainan & Knodel,^2015^). In light of Myanmar having low-quality LTC but also the highest out-of-pocket expenditures for healthcare in Southeast Asia (Teerawichitchainan & Knodel, 2018), we hypothesized that adverse health would predict loneliness and also interact with other variables (e.g., community engagement).

### Present Study

Due to little existing research, the relations between cultural factors (e.g., religion), socio-economic factors (e.g., urban migration, healthcare access), and older people’s mental health in non-WEIRD countries remain unclear. This study aims to help address the knowledge gap about mental health in non-WEIRD countries, with a specific focus on Myanmar. As this is the first study to investigate loneliness in Myanmar’s older population, a mixed-methods approach was used, as it has the powerful advantage of being able to complement quantitative measures with individuals’ reports about their lived experiences (e.g., Haroz et al., 2017; Nguyen et al., 2021). In research on global mental health, it is well-known that quantitative measures of theoretical constructs may not correspond to individuals’ culturally specific understandings of mental health (e.g., depression, loneliness). Thus, qualitative interviews are instrumentally helpful for assessing individuals’ emic experiences of the concepts, in this case loneliness and its predictors. The primary objectives of this mixed-methods study were: (i) to investigate the effect of socio-demographic factors, health, social integration, and religion on loneliness among older people in Myanmar using a national population-based sample; and (ii) to further explore potential underlying mechanisms through qualitative interviews with older adults from southern Myanmar, an understudied and predominantly rural region of the country.

## Subjects and Methods

**Fig. 1:**
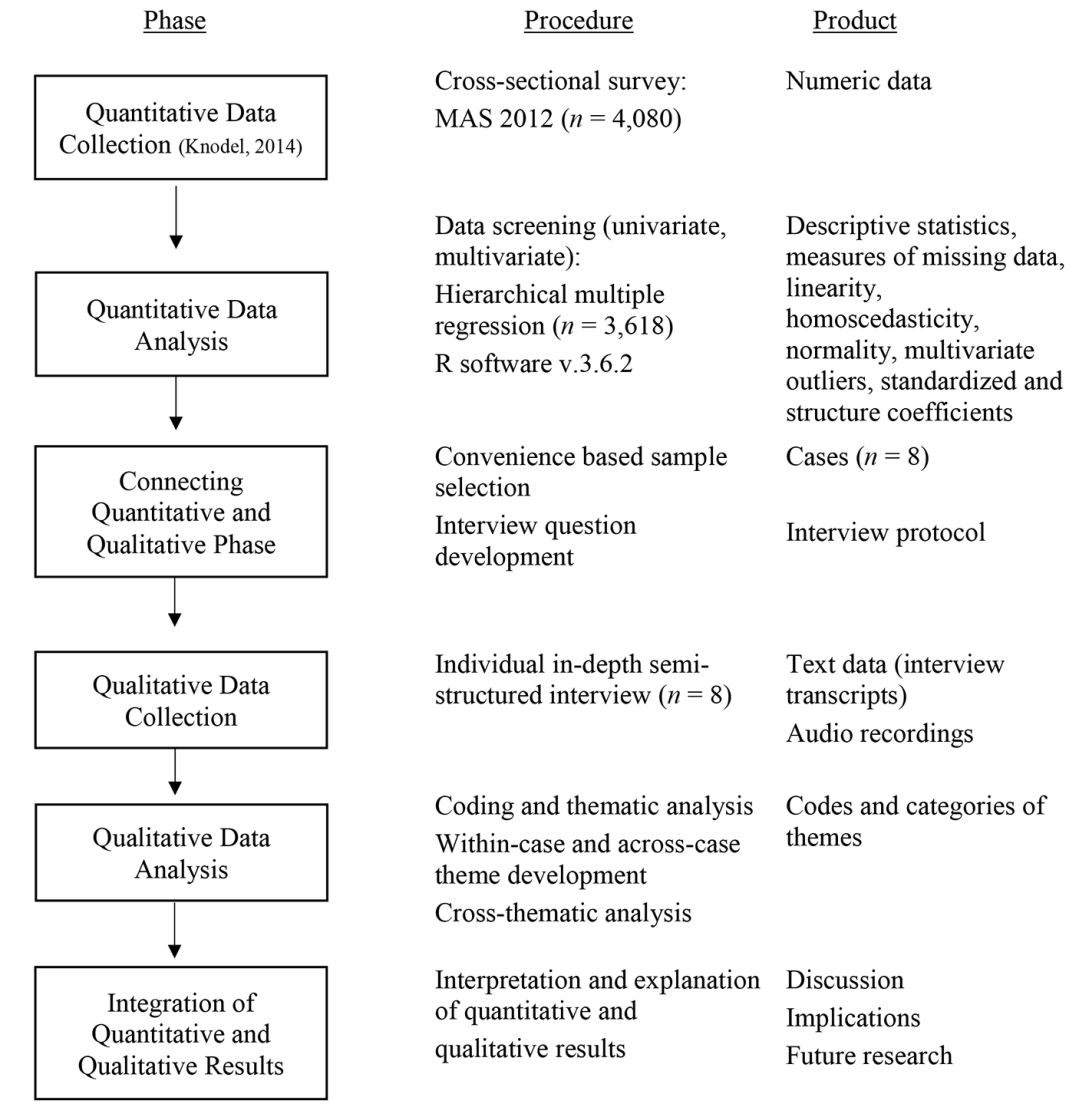
Visual model of sequential explanatory design procedure

Implementing a mixed-methods approach, we combined quantitative with qualitative data in a quantitatively driven sequential explanatory design (Fig.1; Ivankova et al., 2006; Schoonenboom & Johnson, 2017). First, we analyzed quantitative data from a national aging survey conducted in 2012 (Knodel, 2014). Second, we collected qualitative data by conducting interviews that directly built on the quantitative results. As it was not possible to contact participants from the national survey due to data protection reasons, we collected a new sample of older adults for the qualitative interviews seven years later. The aim of this approach was to illustratively explore and complement quantitative findings with comprehensive accounts from qualitative data as to shed light on the understanding of loneliness in Myanmar as well as the potential mechanisms behind the interactions of variables, such as religion, that could not be explained by the quantitative data alone.

### Quantitative Methods and Analysis

#### Procedures and Sample

Quantitative data was obtained from the Survey of Older Persons in Myanmar, commonly referred to as the Myanmar Aging Survey (MAS). The MAS was the first national survey of its kind and was conducted in 2012 under the sponsorship of HelpAge International (Knodel,^2014^). Data consists of 4,080 persons aged 60 and older throughout almost all of Myanmar. The survey included participants from the entire country except for the omission of Kachin state, which was left out due to security reasons. Data was collected through face-to-face interviews using a close-ended questionnaire covering a wide range of aging-relevant topics (for details, see Knodel, 2014; and Teerawichitchainan & Knodel,^2015^).

#### Measures

Loneliness was examined using a single-item self-reported scale: “How often did you feel lonely in the past month?”, with the scores of 1 (not at all), 2 (some of the time), and 3 (often). Although a single item cannot account for the fact that loneliness may have different dimensions, this item has been shown to correlate with measures of loneliness in other population-based studies (Mund et al., 2022).

We included several demographic variables in the analysis, including *age* (in years), *gender* (0 = male, 1 = female), and *education* (1 = no school, 2 = monastic education, 3 = some primary school, 4 = finished primary school, 5 = finished middle school, 6 = vocational, 7 = still in high school, 8 = finished high school, 9 = college/university) to control for possible confounding effects. *Area of residence* (0 = urban; 1 = rural), current *marital status* (0 = no, 1 = yes), and *widowhood* (0 = no, 1 = yes) were coded as dichotomous items. *Economic status* was measured by self-reported total household income per month (on a scale from 1 (< 25,000 MMK^1^) to 5 (> 100,000 MMK)).

*Number of household members*, *having living children* (including adopted children and stepchildren, 0 = no, 1 = yes), receiving social support, and participating in social activities serve as objective indicators for social integration. *Emotional support* was assessed with a single item “Who can you count on to console if you are very unhappy or sad?”. Instrumental support was assessed with the questions “Who helps you with your daily living?” and “Who took care of you during your illnesses or injuries?”. To each of these questions, participants were asked about the people they would turn to for support (e.g., spouse, daughter, son; 0 = no, 1 = yes). The number of people mentioned by participants (1 = yes) was added up to a sum score for each question (range: 0–8), respectively. *Social activities* were indicated by how often in the last year the participant attended community meetings, participated in political meetings or events, socialized with friends and neighbors, did physical exercise in a group, or attended community or religious events. Participants were given a list of these activities and were asked about their frequency of attendance for each social activity. A sum score was created to indicate the total number of social activities in which the participants took part.

*Health status* indexed sum scores of self-reported symptoms from a comprehensive list of 18 conditions that participants had experienced in the past month (multimorbidity; Cronbach’s $\alpha$ = 0.79) and number of limitations in 15 activities of daily living (ADL; Cronbach’s $\alpha$ = 0.66).

*Religion* was assessed with three items indicating importance of religion, as well as frequencies of religious activities at home or at a religious site: “Overall, how important would you say religion is in your life?” (Likert scale from 1 to 4), “In the last month, how often have you gone to the temple (or mosque if Muslim, or church if Christian)?” (Likert scale from 1 to 5), and “How often did you meditate or pray at home during the last month?” (Likert scale from 1 to 5).

#### Statistical Analysis

We applied hierarchical ordinary least squares regression models to examine the role of various predictors on loneliness while accounting for well-known correlates (Cohen et al., 2003). In step 1, we included demographic variables (age, gender, household income, area of residence). In step 2, we included marital status, household members, having children, social activities, and social support. In step 3, we included a model with health indicators (ADL, multimorbidity). In step 4, we included religion. Lastly, in step 5, as aforementioned variables are likely to influence each other (e.g., Quadt et al., 2020), we added interactions and tested interaction effects with the variable of interest. In the final model reported, we retain only those interactions that had emerged as statistically significant with *p <* .05. Examination of the variance inflation factor revealed no multicollinearity among the variables of interest. Listwise deletion was applied for missing values (10.2%). Additionally, 1.2% of participants who reported not knowing the answer to the loneliness question were also excluded from analyses, resulting in a final analytic sample of 3,618 participants. Note that loneliness scores were standardized as t-scores (Mean = 50; SD = 10) prior to analyses.

### Qualitative Methods and Analysis

#### Participants

In-person interviews were conducted in May and June 2019. We used a convenience-based sample, consisting of 8 participants aged 56–96 (M = 82.5) in the areas of Myeik, Tanintharyi region, and Ye, Mon state, located in the coastal zone of southern Myanmar. Both areas, Tanintharyi region and Mon state, are located at the border to Thailand and have some of the highest migration rates to Thailand (Gupta, 2016), suggesting that the prevalence of skipped-generation households may be particularly high in these regions (Teerawichitchainan & Low, 2021). Households in Myanmar’s coastal zones are also more dependent on remittances from migrants to reduce poverty and inequality (Shwe, 2020). Participants were recruited through members of a local nongovernmental organization in Myeik and local English teachers in Ye. As the interviews were not conducted with older adults from the MAS, they mainly serve for illustrative purposes. We also selected participants purposively based on the likelihood of having experienced loneliness in their lifetime, which resulted in a sample consisting of many older old adults. All participants gave oral consent prior to their interviews, which were digitally recorded. The first author and a translator conducted 7 interviews in Burmese, which were later translated into English. The first author’s Burmese language skills allowed her to follow the conversation in Burmese and react accordingly to ensure a natural conversational flow. One interview was conducted in English by the first author only, as the interviewee had fluent English skills. Only the persons transcribing and the authors had access to the interviews.

The interviews were semi-structured with set open-ended introductory questions, taking 40 minutes on average. The main themes in the interview were experiencing and preventing loneliness. The first question was “How do you understand loneliness?”, in order to make sure that loneliness was understood as a subjective state and differentiated from objective social isolation. Further, we asked the interviewees to remember and describe a situation when they had felt lonely before. If they had never felt lonely before, we asked them for potential factors for why they had never felt lonely and why other people felt lonely. Thus, all responses in the results section correspond to questions on experiencing and preventing loneliness. We concluded with some standardized questions about the person’s demographic data, health, social relationships, and religiousness. Ethical principles of informed consent, confidentiality, and avoiding harm were followed rigorously.

#### Qualitative Data Analysis

The data was analyzed to understand the personal experience of loneliness. The interviews were coded using deductive qualitative content analysis (Mayring, 2007). In a first, exploratory step, the first and second authors identified and refined content categories in a third of the responses, following the detailed protocol proposed by Mayring (2007). When the category definitions were sufficiently reliable with Cohen’s Kappa for inter-coder agreement of > 0.70, all remaining responses were coded. The characteristics that participants associated with loneliness were grouped into 9 categories, of which 8 categories were deductively set before coding, and 1 category (“cognition”) was inductively added (see Table2).

## Results

### Quantitative Results

Of the 4,080 participants included in the MAS, 1,960 (48%) participants were between 60 and 69 years old, whereas 2,120 (52%) were over 70 years old. A majority of participants identified as Buddhist (95%) and ethnic Bamar (71%), followed by Rakhine (8%), Kayin (7%), and Shan (6%) ethnic minorities. Over three-fourths of older people prayed or meditated daily during the past month. The average household size of older people was 4.6; only 7% lived in single person households, whereas 10% lived in households with more than eight members. 95% of older people had at least one child who either lived with them, next door, or in the same community. Two thirds of adult children provided parents with some material support during the past year and, particularly daughters, played a prominent role in helping older parents with their ADL. Only one-third of older people completed formal primary school or higher education and almost a third of participants worked in the past year. Most older people in Myanmar live in abject poverty, with only half of the MAS sample indicating that their income was adequate to meet their daily needs and a majority not earning more than US$3 per day. The overall response rate was 92.6%; only 0.6% refused to take part in the interview. Detailed sample characteristics of the MAS have been described elsewhere (Knodel, 2014; and Teerawichitchainan & Knodel, 2015).

Among the final analytical sample of 3,618 participants, 2,471 (68.3%) of participants reported never feeling lonely, 952 (26.3%) felt lonely some of the time, and 195 (5.4%) felt lonely often. Consistent with previous research, loneliness was associated with being older, being female, not being married, being widowed, having fewer household members, not having children, having lower household income, participating in fewer social activities, receiving less emotional support, having more ADL limitations, and having higher multimorbidity (Supplementary Table S-2). Interestingly, higher loneliness was associated with practicing more religious activities at home.

#### Predictors of Loneliness in Myanmar’s Older Population

Table 1 presents results from hierarchical regression analyses. Findings revealed that when all variables had been included (Model 4), having lower household income, being widowed, having fewer household members, not having children, participating in fewer social activities, suffering from more ADL limitations, multimorbidity, and practicing more religious activities at home were each associated with higher loneliness. In total, the effect size amounts to *d* = 0.20, with R^2^ = 0.12.

Most demographic variables, such as age, gender, education, and area of residence did not reveal associations with loneliness (*p* > .05). However, loneliness was related to having lower household income (ß = − 0.06, *p* < .001) and living with fewer household members (ß = − 0.11, *p* < .001). Although being married itself had no significant predictive effect on loneliness in the regression (*p* > .05), being widowed (ß = 0.14, *p <* .001) significantly increased the likelihood of feeling lonely. People with living children were less likely to feel lonely (ß = − 0.07, *p* < .001) than people without living children. The main effect of social activities was negatively associated with loneliness indicating that people who frequently participated in social activities were less likely to feel lonely (ß = − 0.06, *p* < .001). Against our expectations, there were no significant associations of receiving emotional or instrumental support with loneliness (*p* > .05).

As hypothesized, older adults with multimorbidity (ß = 0.07, *p* < .001) or a higher number of ADL limitations (ß = 0.09, *p* < .001) were more likely to feel lonely. Interestingly, different aspects of religion had different associations with loneliness. Neither religious activity engagement outside or religious importance were related to loneliness in the MAS (*p* > .05). However, practicing more religious activities at home (ß = 0.05, *p* < .01) was linked to higher loneliness.

**Table 1:** Standardized prediction effects (ß) from regression analysis with loneliness as outcome (n = 3,618)

Predictors			Loneliness							
			Model 1	Model 2			Model 3		Model 4	Model 5
Age			0.08***	0.02			–0.03		–0.03	–0.02
Gender			0.13***	0.03			0.02		0.02	–0.07
Education			–0.04	–0.04*			–0.03		–0.03	–0.04
Household Income			–0.13***	–0.07***			–0.06***		–0.06***	–0.06***
Area			–0.02	–0.01			–0.02		–0.02	–0.01
Married			–	–0.06			–0.06		–0.07	–0.06
Widowed			–	0.14***			0.15***		0.14***	0.15***
Household Members			–	–0.10***			–0.11***		–0.11***	–0.46**
Children			–	–0.07***			–0.07***		–0.07***	–0.07***
Social Activities			–	–0.06***			–0.05**		–0.06***	0.22**
Emotional Support			–	0.00			–0.01		–0.01	–0.01
Instrumental Support			–	0.00			0.01		0.01	0.02
ADL limitations			–	–			0.09***		0.09***	0.16***
Multimorbidity			–	–			0.07***		0.07***	0.07***
Religious Activity			–	–			–		0.03	–0.02
Religious at Home			–	–			–		0.05**	0.28***
Religious Importance			–	–			–		–0.01	–0.09*
Gender x Religious Activity			–	–			–		–	0.10*
Household Members x ADL limitations			–	–			–		–	–0.09*
Household Members x Religious Importance			–	–			–		–	0.40**
Social Activities x Religious at Home			–	–			–		–	–0.40***
Total *R*^*2*^			0.051	0.097			0.110		0.112	0.121
F	41.72***				26.99***	25.90***		3.50*		8.62***
(*df1, df2*)		(5;3,612)		(7; 3,605)			(2; 3,603)		(3; 3,600)	(4; 3,596)

*Note*. Results shown in this table are unweighted. Gender: 0 = male; 1 = female; Education: 1 = no school; 2 = monastic education; 3 = some primary school; 4 = finished primary school; 5 = finished middle school; 6 = vocational; 7 = still in high school; 8 = finished high school; 9 = college/university, Area: 0 = urban; 1 = rural, Married: 0 = not married; 1 = married, Widowhood: 0 = not widowed; 1 = widowed, Children: 0 = no, 1 = yes. *p < .05, ** *p* < .01, *** *p* < .001.

#### Interactions between Gender, Religion, Social Activities, Health, and Financial Situation

**Fig. 2:**
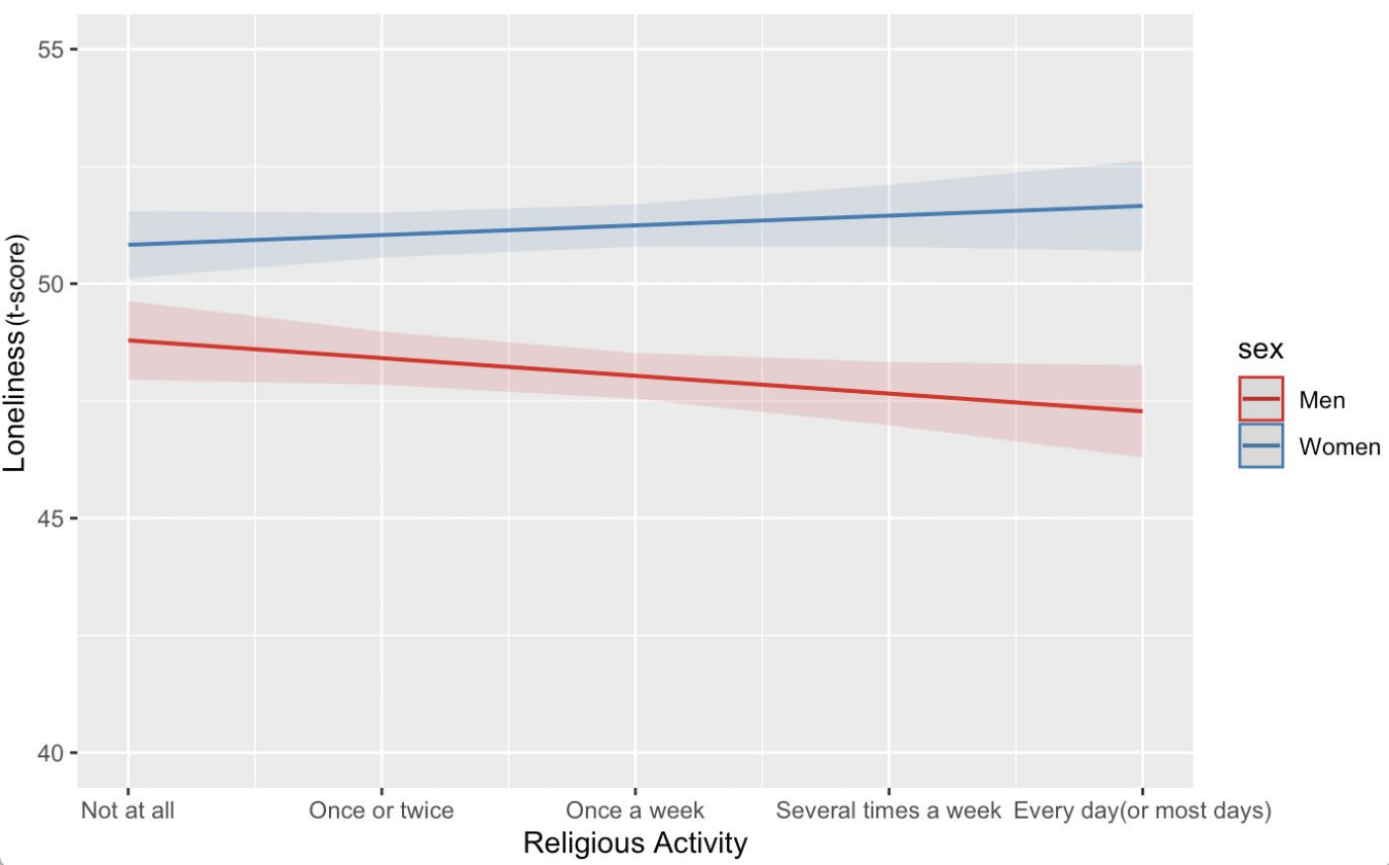
Interaction effects between gender and religious activity on loneliness. Significant interaction effects with gender and religious activity indicate that men reported considerably less feelings of loneliness when they frequently participated in religious activities. But among women, no differences in loneliness were observed in regard to participating in religious activities. The blue line represents women and the red line represents men.

**Fig. 3:**
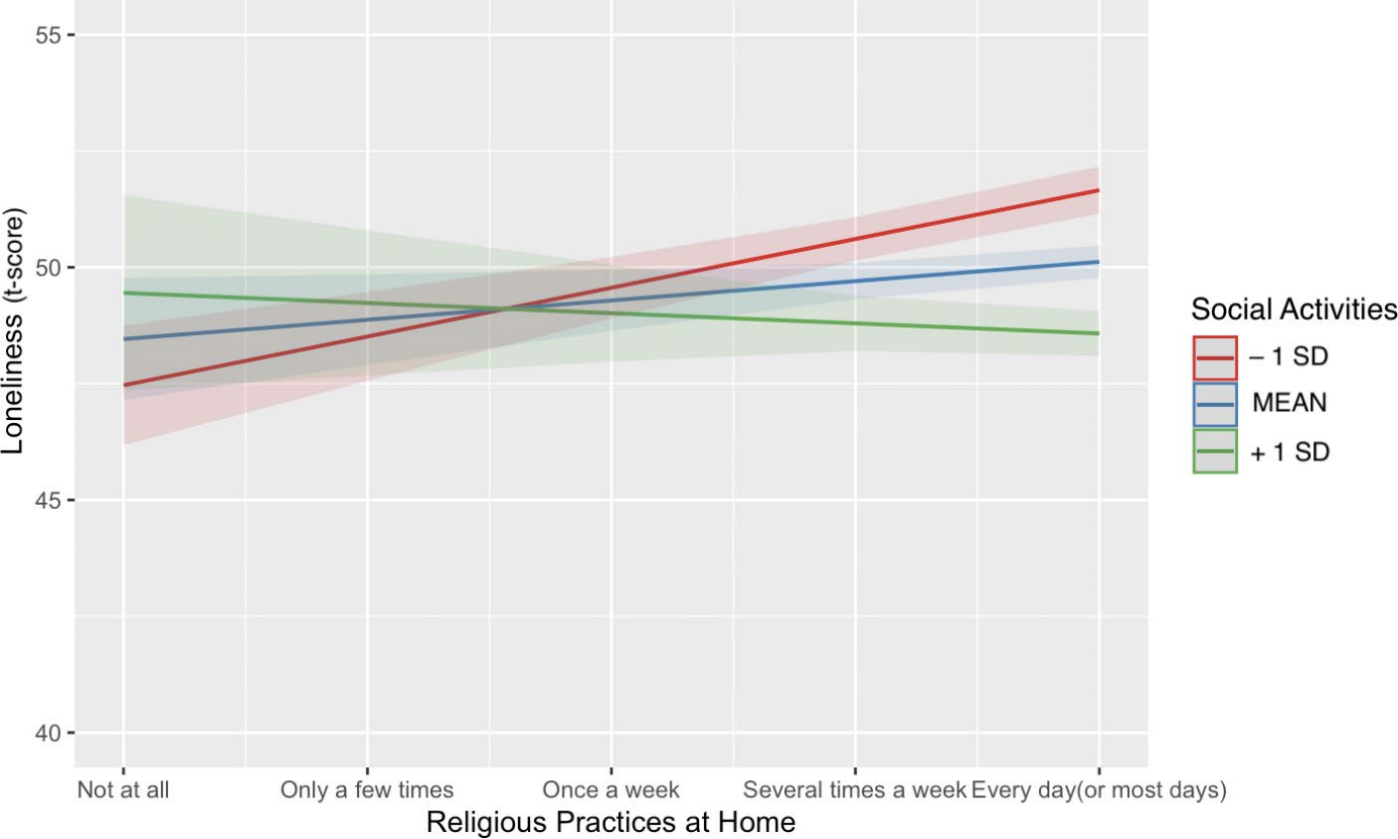
Interaction effects between social activities and religious practices at home on loneliness. Significant interaction effects with social activities and religious practices at home indicate that people who did not participate in social activities reported considerably higher feelings of loneliness when they prayed or meditated at home. But older people who participated in many social activities reported less feelings of loneliness when they frequently prayed or meditated at home. The blue line indicates average social activity participation, the green line indicates one standard deviation above average, and the red line indicates one standard deviation below average. Note that social activities was used as a continuous variable in our analyses and categorized into three groups for graphical illustration only.

Our regression analysis indicated several interaction effects (Model 5). First, we found a significant interaction between gender and religious activity (ß = 0.10, p < .05): Men reported being less lonely when frequently participating in religious activities, whereas this was not the case for women (Fig.2). Second, although religious importance had no main effect on loneliness, number of household members moderated the association of religious importance with loneliness. Participants who reported religion to be less important and had more household members reported less loneliness (ß = 0.40, *p* < .01). In contrast, people who perceived religion to be very important did not report differences in loneliness in regard to household members. In a similar vein, Fig.3 illustrates the significant interaction between social activities and religious practices at home (ß = − 0.40, *p* < .001). For older adults who did participate in social activities, religious practices at home (e.g., meditating, praying) predicted lower loneliness. But for older adults who did not participate in social activities, religious practices at home were associated with higher loneliness. Finally, number of household members interacted with ADL limitations (ß = − 0.09, *p* < .05): For people with few household members, ADL limitations predicted greater loneliness. But for people with many household members, ADL limitations did not predict loneliness.

### Qualitative Results

#### Loneliness in Southern Myanmar

When asking participants about the understanding of loneliness, most participants described an emotional reaction to social rejection and referred to the desire of being loved and cared for. Some participants also associated loneliness with being alone but then gave examples of feeling lonely when being with other people, indicating that loneliness is understood as distinct from social isolation.

*“Loneliness is when you don’t have relationships (…) and when nobody cares for you, it makes you weak, or a few people.”* – Participant 3 (Female, 74, Burmese, Muslim).

Table 2 presents interviewees’ answers to the question: “In which situations do you usually feel lonely?”. If interviewees had never felt lonely, they gave possible explanations for why they had never felt lonely. Thus, participants’ responses highlighted either risk factors or protective factors of loneliness. Summing across both kinds of responses, the most frequently mentioned themes were *Family (15)*, followed by *Financial Situation (7), Relationship Quality (6)*, and *Health (5).* In the interviews with older adults in Tanintharyi region and Mon state, 2 of the 8 interviewees reported that they had never felt lonely, while 6 interviewees reported having felt lonely before. Of these 6, 4 interviewees reported currently feeling lonely. Interviewees who currently felt lonely were either widowed (*n* = 3) or never married (*n* = 1), whereas both interviewees who did not currently feel lonely were married (characteristics in Supplementary Table S-1).

**Table 2:** Categories and examples of predictors (risk factors; protective factors) for loneliness

Categories (Frequency)	Examples
Family (15)	**Risk factor**: Seeing other families and realizing that one’s own family is not there or has passed away. **Protective factor**: Living together with a family protects from being lonely, as one is always busy and surrounded by people who can support the older person.
Financial Situation (7)	**Risk factor**: Worrying about where to get food from, especially when the responsibility lies upon one person. **Protective factor**: Not needing to worry about food and living protects from negative feelings, such as loneliness.
Relationship Quality (6)	**Risk factor**: When people do not care about how you feel and leave you alone because you are old. **Protective factor**: Being satisfied with close relationships is specifically important to not feeling lonely.
Health (5)	**Risk factor**: Not being healthy means being immobile and not able to do anything or participate in activities. It also makes you dependent on other people to take care of you. **Protective factor**: Being healthy enables independence, like doing household work without needing any help from others.
Community Activity (2)	**Risk factor**: Realizing one is alone at community activities, whereas other families and people come together. **Protective factor**: Participating in community activities leaves no time to think about loneliness.
Cognition (2)	**Risk factor**: Being too proud to ask other people for help makes you not care about other people’s opinions. **Protective factor**: Having a good attitude by keeping a smile when other people say rude words in front of you.
Area of Residence (2)	**Risk factor**: Living in a big city can be a big burden for old people as it becomes harder to find intimate social contacts and be mobile or participate in community activities. **Protective factor**: In cities, water comes from a pipe and can facilitate taking a bath, compared to a well in the village. People in a village are dependent on support by family members and may feel lonely when they do not have a person to support them.
Instrumental Support (2)	**Risk factor**: n.a. **Protective factor**: Children or neighbors take care of you in case of illness or needing help. Likewise, providing care for children when they need something can also prevent loneliness.
Emotional Support (1)	**Risk factor**: When nobody cares for you, it makes you feel weak and lonely. **Protective factor**: n.a.

**Social determinants.** Although the quantitative results did not show differences in loneliness between residents of urban versus rural areas, Participant 2 mentioned living area in response to being asked about loneliness (Supplementary Table S-3). Importantly, financial situation was one of the strongest themes that emerged when asking interviewees about situations in which they felt lonely:

*“Before, I was rich, and there were many people around me. But now, I am poor, and nobody cares about me (…). Before, I had money, I gave my children everything. After they got the money, they didn’t care about me anymore. (…) When I didn’t have money, I felt lonely. Nobody cares for me. Really.”* – Participant 4 (Female, 91, Burmese, Buddhist).

**Family.** In line with our hypothesis, “family” was the theme most associated with feeling lonely in the interviews and was mentioned as a protective factor and a risk factor for loneliness. According to several interviewees––and in line with our hypothesis––having children was more protective against loneliness than marital status. However, as many children cease to live with their parents after getting married, their absence may also be a risk factor for feeling lonely.

*“People who are lonely don’t have a perfect life, unlike people who have a family. (…) I feel lonely and unhappy of course. (…) Whoever, rich or poor, if a person has a family, their life is perfect.”* – Participant 1 (Female, 56, Burmese, Hindu).

*“Even when you marry, but don’t have children, you will be lonely. Find a family.”* –Participant 3 (Female, 74, Burmese, Muslim).

*“I have one son, he left 8 years ago. (…) I miss him, I don’t feel well, because I miss him every day.”* – Participant 5 (Female, 80, Mon, Buddhist).

*“My family was in Myeik, and I always thought about how long I will stay in Yangon and when I will transfer back to Myeik. (…) I felt lonely and missed my family. [Now] I live with my family together, so I don’t have any worries, I am not alone. Everyone speaks to me, we have conversations. Very easy, no worries.”* – Participant 8 (Male, 93, Burmese, Muslim).

Beyond just the quantity of family members, the quality of relationships was also strongly associated with loneliness in the interviews, lending support to the distinction between subjective loneliness and objective social isolation. Relationship quality also varied across the lifespan, likely due to older adults’ social status (see Participant 6).

*“When there are many people, and they might go outside and not care about me, then I’m left alone and feel lonely.”* – Participant 8 (Male, 93, Burmese, Muslim).

*“Everyone blames you, that’s loneliness. I do everything right, but someone always uses me, nobody appreciates me, doesn’t say thank you. No one respects what I do for other people. (…) I have [felt lonely], I want people to take care of me and love me. (…) When I was young, everyone wanted me, but now, nobody wants me.”* – Participant 6 (Male, 96, Burmese, Muslim).

*“I’m never disappointed because of my children. I don’t need to tell them what I need, because I already have everything. There is no loneliness, never. There is only happiness. My situation is perfect.”* – Participant 7 (Female, 79, Mon, Buddhist).

**Community and social support.** In line with the quantitative results, Participant 3— who never felt lonely before—reported being very active in her community and not having time to feel lonely (Supplementary Table S-3). From another perspective, social activities can also evoke feelings of loneliness, potentially exacerbated by social comparisons. Participant 1, who often felt lonely and wished to have a family, reported feeling lonely especially at community activities. Although the quantitative results did not find any relations between social support and loneliness, our qualitative findings suggested otherwise.

*“When there is a festival, I feel that I am alone and don’t have anyone. When I see other family members, I really want to be part of that.”* – Participant 1 (Female, 56, Burmese, Hindu).

*“I get such good support from my neighbors, which has made it possible for me to live alone for over 30 years now. They take care of me like a family. When I am sick, they come to my home and take care of me.”* – Participant 1 (Female, 56, Burmese, Hindu).

**Health.** Impaired health was––consistent with the quantitative analysis––strongly associated with loneliness in the interviews. When participants were asked about advice on how to protect oneself from loneliness, most interviewees referred to the importance of being healthy. Three interviewees also reported that adverse health was a consequence of feeling lonely.

*“If you are with many people, or lonely, in any case, the most important thing is to be healthy. Not to get an illness is very important, that is the main thing, to be healthy. If you are not healthy, you cannot do anything.”* – Participant 8 (Male, 93, Burmese, Muslim).

*“If we don’t reduce loneliness, we will become sick. Now, I have a heart disease.”* – Participant 1 (Female, 56, Burmese, Hindu).

**Interactions and underlying mechanisms.** We used the results of the qualitative study to shed light on the quantitative interaction results. Participant 3 reported that being religious motivated her to participate in community activities and be socially integrated, thus protecting her from loneliness. However, Participant 4, who felt lonely, explained that she could only engage in religious practices at home due to underlying health constraints, which makes sense of the interaction that showed that religious activity at home predicted higher loneliness for people unable to attend social activities. It is worth noting that health constraints may not only be directly associated with loneliness but also be a major factor that keeps people from attending community activities, in turn leading them to only practice religious activities at home as well as feel lonely.

Also, recall that ADL limitations interacted with number of household members. Relevant to the finding that in people with few household members, ADL limitations predicted greater loneliness, Participant 1, who lives alone, expressed her worries of becoming ill and not being able to work anymore. This interaction may be specifically relevant to older people living in rural areas who are dependent on their household members to support them with their ADL, e.g., like taking a bath at the well.

*“The prophets say that humans should live in the community and not live alone. (…) Allah really loves me, that’s why he made me poor. I thank Allah for that. I’m really glad. I always pray to Allah not to have poor health, but I don’t want to be rich, because I want to do more religious work for Allah.”* – Participant 3 (Female, 74, Burmese, Muslim).

*“Before, I always meditated. I cannot meditate anymore, because I need to go to the toilet. I wanted to meditate in a monastery for 3 months, but after 2 weeks I needed to go home because I couldn’t eat and sleep anymore. (…) Every night I pray at home.”* – Participant 4 (Female, 91, Burmese, Buddhist).

*“I am healthy, so I have work. It’s very important for me to stay healthy, because if I’m healthy I can work and care for myself. I am afraid to become sick, then I cannot take care anymore.”* – Participant 1 (Female, 56, Burmese, Hindu).

*“Here, in Myeik, the water comes from a pipe, so it’s easy to take a bath. In the village, where I live, there is a well, so it’s not possible for me to bathe myself.”* – Participant 4 (Female, 91, Burmese, Buddhist).

Another striking finding from the qualitative data––going beyond quantitative results––shed light on the important link between financial insecurity and community engagement, religion, and health for loneliness. Note that all interviewees who mentioned their financial situation in regard to loneliness were female. Participant 5 said she was unable to participate in the community due to financial issues, and she also referred to herself as being “too old to help”. She further described that she does not have money to pay for the medicine she needs to be healthy and walk without pain, which are undoubtedly also challenges to community participation. Nevertheless, Participant 1 and Participant 3 expressed that having a good social environment and being healthy were more important than their financial situation for participating in their community and preventing loneliness.

*“I don’t have money to participate in community causes. I cannot give them money when they ask me. I am too old to help them with their work.”* – Participant 5 (Female, 80, Mon, Buddhist).

*“It is very important to have a good environment. If I had the option to live in a different place, a silent place where there were no people but where I could become very rich, that would be worse for me.”* – Participant 1 (Female, 56, Burmese, Hindu).

## Discussion

The goal of the present study was to examine the experience and predictors of loneliness for older people in Myanmar. As shown in our interviews with older adults in southern Myanmar, loneliness was understood as distinct from social isolation, which is in line with common definitions of loneliness in other countries. A recent study found that there were no fundamental differences in defining loneliness in both socially embedded and less embedded cultures (Heu et al., 2021). Findings from both the quantitative and qualitative data revealed that widowhood, lower number of household members, not having children, poorer financial situation, poorer health, fewer social activities, and more religious activity at home were each directly associated with higher loneliness. Effect sizes were in the small to moderate range (Hedges, 2008). In addition to highlighting diverging findings, we will discuss underlying mechanisms of loneliness predictors.

First, we did not find associations of gender, age, or marital status with loneliness. Previous work (Dahlberg et al., 2018) suggests that it is not age and gender per se, but widowhood or greater levels of health problems among the oldest old that are associated with loneliness. Similarly, widowhood was a consistent predictor for loneliness in both datasets. Additional reasons for why being married per se had no effect on loneliness may be understood by looking at the country context. Unlike in neighboring Thailand, very few older people actually live alone in Myanmar (Knodel & Pothisiri, 2014), and older unmarried adults (a pyo kyi and lu pyo kyi) usually reside with a sibling’s family. Although these unmarried adults may feel lonely due to not having a spouse in the first place (especially at a younger age), they do not experience the loss of a partner in old age. Bereaved people, however, experience a sudden change in their family relationships due to the death of their spouse—and a stark discrepancy between their desired and actual relationships (Bennett et al., 2019). A lifespan approach with longitudinal data is needed to further understand the development of loneliness in the sub-populations of unmarried versus widowed adults in Myanmar.

Further, our results indicated that lower household income was associated with higher loneliness in both the quantitative and qualitative findings. Interviewees listed reasons for feeling lonely that were related to their financial situation, such as food security, not being able to buy medicine, not being able to contribute to community activities, and undermined social status as a result of poverty. The exacerbating effect of financial situation on loneliness is in line with previous literature (Cohen-Mansfield et al., 2016; von Soest et al., 2018). Interestingly, only women from our qualitative sample alluded to financial problems associated with loneliness, which accords with findings that older women are especially vulnerable to financial insecurity in Myanmar (Lerdsrisuntad, Srisilapanan, Kaewkantha, Pussayapibul, & Toh, 2018). Our findings jointly indicate that financial security may serve as a tool for being integrated in the community and protect from loneliness (e.g., by ensuring physical health), but that being rich is not as important as having “a good environment” of people who care about you.

Accordingly, social activity engagement was related to loneliness in both the qualitative and quantitative findings, consistent with previous findings (Carr et al., 2018). While quantitative data suggested that higher social activity engagement predicted lower loneliness, qualitative data showed that the association is more heterogeneous. Based on the interviews, participation in community activities is not exclusively a protective factor but may also evoke feelings of loneliness due to social comparisons, family context, financial situation, or adverse health. These findings may shed light on why previous intervention programs that encouraged older people to participate in community activities were not very successful at alleviating loneliness (Masi et al., 2011).

Next, having living children was associated with less loneliness. Here, the qualitative data generated insights that the quantitative data alone could not reveal. The quantitative analysis did not consider the relationship quality with children or family members living in the same household. Our qualitative findings suggested that the mere number of family members is not predictive of loneliness (Cohen-Mansfield et al., 2016). Rather, it is the physical presence of family members, such as romantic partners and children, at home—as well as the relationship quality with these family members—that predicts loneliness. Further, interviewees highlighted the role of neighbors as source of support, which may be especially relevant to older people’s mental health in non-WEIRD contexts (Ojagbemi & Gureje, 2019).

Impaired health was strongly associated with loneliness and other negative outcomes, such as decreased autonomy and increased dependence on caregivers. One common theme from the interviews was that impaired health hindered older people from participating in community activities. Our findings showed that social support (e.g., by household members) may buffer against the negative effects of adverse health on loneliness, especially in rural areas where, for instance, older adults depend on someone else getting water for them from the well. Our findings further allude to the entangled relationship between health and loneliness (Ong et al., 2016), highlighting that adverse health is not only a potential antecedent of loneliness but also an outcome. Given that the effect of loneliness on health appears to be even stronger in more collectivistic countries (Beller & Wagner, 2020), our findings underline that physical proximity and satisfying relationships with family members are essential to ensure older people’s physical and mental well-being.

Relationship quality appeared as a strong theme in the interviews. Several interviewees mentioned feeling neglected, not appreciated, not cared about, or even left alone by their family members. These findings reflect the definition of loneliness (Peplau & Perlman, 1982) and accord with previous literature distinguishing between social isolation and loneliness on adverse health outcomes (Bu et al., 2020; Rafnsson et al., 2020). Interestingly, interviewees also mentioned their age as a reason they were not being cared about. As we did not find a relation between chronological age and loneliness in our regression analysis, the individuals’ perception that their age was linked to their loneliness may have to do with larger societal trends. Myanmar’s rapid cultural and economic changes may be leading to changes in norms concerning the standing of older adults (HelpAge International, 2019; Lerdsrisuntad et al., 2018), with ageist stereotypes potentially becoming more prevalent and creating new pathways to loneliness (e.g., Shiovitz-Ezra et al., 2018).

Finally, an extremely influential element of Myanmar’s culture is religion. Religion is known to be a powerful influence on people’s behaviors, beliefs, and coping mechanisms (Norenzayan et al., 2014; Rokach 2018). The interviews showed that religious considerations motivated social engagement in community activities and also had an effect on people’s mindsets. Nevertheless, our results do not entirely support previous findings—based on other samples, such as Christian Romanian migrants—that religious activity protects against loneliness (Ciobanu & Fokkema, 2017). Rather, our findings are line with a recent study by Warner and colleagues (2019), who showed that the association of religion and loneliness may be mediated by complex interactions with demographic factors. Our integrated results also showed that people who meditated or prayed at home were more likely to feel lonely. This result may be explained by the assumption that meditation may in fact be a collective practice that people enjoy doing together (e.g., in meditation retreats), and miss when they have to stay home due to health constraints, resulting in feelings of loneliness.

Our study has several limitations. First, we observed little variability in the importance of religion, which may have altered our findings in quantitative analysis. Further, the qualitative sample mainly consisted of individuals aged 74 years and older, which can be considered as the older old group in Myanmar, as these participants reached an age above average life expectancy (Knodel, 2014). Consequently, those individuals may be dealing with different challenges than the average aged older adult in Myanmar, indicating a selection bias. Although our qualitative sample may not be representative of the average older person, the aim of our convenience-based sample was to shed light on the experience of loneliness in Myanmar, which is why participants were not selected based on chronological age but on the likelihood of having experienced loneliness in their lifetime. Future studies may need to include more younger old adults in qualitative studies to investigate potential differences in loneliness among the heterogeneous aging population. Second, our study was cross-sectional, and we could not follow up with participants from the MAS in our qualitative sample, which led to the qualitative data being collected seven years after the quantitative data. Thus, we could not investigate the causality and direction of associations. Third, in our regression models, we only included the number of children and did not account for whether children co-resided with their parents. Given that living arrangements in the MAS have been previously studied in detail (Teerawichitchainan et al., 2015), and that 83% of older adults with children also co-resided with them, suggests that including this variable would not have changed our study’s conclusions. Moreover, by only measuring loneliness once, we could not distinguish between transient and persistent loneliness and its associated predictors and outcomes on an intra-individual level (e.g., Vingeliene et al., 2019). The need to distinguish between them is reinforced by our qualitative findings, which showed clearly how loneliness is context- and situation-dependent and changes over time (Akhter-Khan et al., in press).

Although participants seemed to talk openly about loneliness in the interviews, we are aware that cultural concepts like “anade”^2^––a reluctance to discuss needs for fear of being imposing or burdensome (Bekker, 1981) ––may have biased the older peoples’ answers and thus led to an underestimation of the prevalence or severity of loneliness. This also suggests that measuring loneliness with a single item may not be sufficient for identifying different dimensions of loneliness that may be targeted by different interventions (Akhter-Khan & Au, 2020; Gierveld & van Tilburg 2006). Finally, our data from 2012 did not allow us to explore the association between technology and loneliness. The impact of technology on older people’s lives, as well as its interaction with culture-specific concepts like “anade”, are exciting avenues for future research.

### Implications

Myanmar’s older people, specifically those facing bereavement and financial distress, are at risk of feeling lonely. Our results suggest that loneliness and health are not age-dependent and indicate a need to move away from age-oriented policies and towards need-oriented support policies, particularly for older women. Microfinance projects may be scaled up while enhancing their access for women who are facing inequalities in regard to highly gendered care, educational, and work roles in Myanmar’s society, which contribute to financial insecurity (Lerdsrisuntad et al., 2018) and may result in serious health risks (Teerawichitchainan & Knodel, 2015). Moreover, the value and reach of pension payments are still very limited and urgently need to be expanded, as pension payments currently only reach older adults who are 85 years and older and have national registration cards (Knodel & Teerawichitchainan, 2017; Yamada et al., 2019).

Given that community engagement has different effects on loneliness depending on individual contexts—in some cases even increasing loneliness due to social comparisons—it is important to not suggest “one size fits all” programs when implementing interventions globally. Rather, it is necessary to develop individually tailored programs that fit the health, family, and motivational context of the person (Akhter-Khan et al., in press; Fakoya et al., 2020). With this approach, also known as “precision health”, interventions may prevent chronic loneliness and related diseases using the right solution, for the right person, at the right time (Akhter-Khan & Au, 2020). Considering the rapid pace of political and economic changes in Myanmar, it becomes even more important to tailor interventions to individuals’ social and cultural contexts. In Myanmar, older people’s self-help groups are one example of how older people’s social, economic, and health needs can be met holistically through community programs (Knodel & Teerawichitchainan, 2017). Whether these inclusive self-help groups successfully prevent loneliness (especially for those who are most vulnerable due to impaired health) remains to be investigated.

Finally, mental health programs in Myanmar are insufficient (Nguyen et al., 2018) and may benefit from adopting the kind of lay-care approaches used in other non-WEIRD countries that are coordinated with local community organizations (e.g., Patel et al., 2017). Religious leaders have substantial reach, including to high-risk groups, and could act as lay-care counsellors for mental health issues in Myanmar and similar socio-economic contexts. Programs like Zimbabwe Friendship Benches (Chibanda et al., 2017) may increase possibilities for older women to take on leadership roles in the community and be socially integrated and respected. Due to pervasive stereotypes about mental health and threats arising from ageism, intergenerational community mental health interventions potentially open up new possibilities to address loneliness in older populations. We hope that our study will motivate future research on loneliness and mental health interventions in Myanmar. Only then will it be possible to fully grasp the heterogeneity of loneliness and ensure well-being for older adults in Myanmar and globally.

## Electronic supplementary material

Below is the link to the electronic supplementary material.

Supplementary Material 1

## Footnotes

1 25,000 Myanmar Kyat (MMK) was equal to approximately US$30 in 2012.

2 Anade is a concept inherent to understanding Myanmar’s culture and social relationships, indicating politeness and the value of not putting a burden on someone by sharing ones worries or by initiating care provision by other people. Although there is no direct translation to English, it is a concept comparable to “Kreng Jai” in neighboring Thailand, which has been covered more widely in research (e.g., Areemit et al., 2021).
