# Supplementary material for: Loneliness in Myanmar’s older population: A mixed-methods investigation
Source: J Cross Cult Gerontol. 2022 Oct 27;37(3):315–37. doi: 10.1007/s10823-022-09459-x (PMC9643263; doi:10.1007/s10823-022-09459-x)
Supplement: Supplementary file 1 — Supplementary Material 1 [file 10823_2022_9459_MOESM1_ESM.docx]

**Supplementary Material**

**Table S-1***.* Interviewee Characteristics (at the time of interview), (n = 8)

| Participant | Age | Gender | Relationship Status | Household Members^1^ | Ethnicity /  Religion | Residence | Loneliness^2^ |
| --- | --- | --- | --- | --- | --- | --- | --- |
| Participant 1 | 56 | F | Never married | 1 | Burmese / Hindu | Myeik | Yes |
| Participant 2 | 91 | M | Married^3^ | 8 | Burmese / Christian | Myeik | No |
| Participant 3 | 74 | F | Married | 18 | Burmese / Muslim | Myeik | Never |
| Participant 4 | 91 | F | Widowed | 5 | Burmese / Buddhist | Village^4^ | Yes |
| Participant 5 | 80 | F | Widowed | 2 | Mon / Buddhist | Ye | Yes |
| Participant 6 | 96 | M | Widowed | 5 | Burmese / Muslim | Myeik | Yes |
| Participant 7 | 79 | F | Married | 4 | Mon / Buddhist | Ye | Never |
| Participant 8 | 93 | M | Married | 15 | Burmese / Muslim | Myeik | No |

*Note.* ^1^Household members including self. ^2^“Never” people who never experienced loneliness before; “No” people who experienced loneliness before, but currently do not feel lonely; “Yes” people who currently feel lonely. ^3^Although married, Participant 2 and his wife lived apart due to her work in another city. ^4^ Participant 4’s village was 35 minutes away from Myeik.

**Table S-2.** Intercorrelations (Part 1) (n = 3,618)

|  | Intercorrelations | | | | | | | | | | | |  |
| --- | --- | --- | --- | --- | --- | --- | --- | --- | --- | --- | --- | --- | --- |
| Variables (range) | M (SD) | 1 | 2 | 3 | 4 | 5 | 6 | 7 | 8 | 9 | 10 |  |  |
| 1. Loneliness (1-100) | 49.8 (9.9) |  |  |  |  |  |  |  |  |  |  |  | |
| 2. Age (60–104) | 70.4 (7.6) | 0.11** |  |  |  |  |  |  |  |  |  |  | |
| 3. Gender (0 men, 1 women) | 0.56 (0.5) | 0.15** | 0.07** |  |  |  |  |  |  |  |  |  | |
| 4. Education (1–9) | 3.0 (2.0) | –0.10** | −0.21** | −0.22** |  |  |  |  |  |  |  |  | |
| 5. Household Income (1–5) | 3.0 (1.3) | –0.14** | 0.04* | –0.05** | 0.19** |  |  |  |  |  |  |  | |
| 6. Area (0 urban; 1 rural) | 0.7 (0.5) | 0.01 | –0.06** | –0.03 | −0.35** | −0.20** |  |  |  |  |  |  | |
| 7. Married (0 no; 1 yes) | 0.4 (0.5) | –0.25** | –0.30** | –0.45** | 0.19** | 0.10** | 0.00 |  |  |  |  |  | |
| 8. Widowed (0 no; 1 yes) | 0.5 (0.5) | 0.24** | 0.35** | 0.41** | –0.21** | –0.08** | 0.01 | –0.88** |  |  |  |  | |
| 9. Household Members (1–16) | 4.6 (2.4) | −0.17** | −0.04* | –0.09** | 0.02 | 0.38** | –0.04* | 0.14** | –0.10** |  |  |  | |
| 10. Children (0 no; 1 yes) | 0.9 (0.2) | –0.07** | 0.06** | –0.06** | –0.05** | 0.10** | 0.02 | 0.14** | 0.15** | 0.14** |  |  | |
| 11. Social Activities (0.00–21.00) | 9.8 (2.3) | −0.11** | −0.20** | –0.17** | 0.07** | 0.10** | 0.06** | 0.15** | –0.15** | 0.04* | 0.00 |  | |
| 12. Emotional Support (0.00–6.00) | 1.3 (1.0) | −0.08** | 0.01 | –0.05** | 0.04* | 0.19** | 0.04* | 0.17** | −0.12** | 0.19** | 0.11** |  |  |
| 13. Instrumental Support (0.00–4.00) | 1.4 (0.5) | 0.00 | –0.08** | –0.02 | 0.05** | –0.07** | –0.01 | 0.05** | –0.05** | –0.06** | 0.01 |  |  |
| 14. ADL limitations (0.00–24.00) | 5.1 (4.7) | 0.15** | 0.43** | 0.14** | –0.23** | –0.06** | 0.05** | –0.20** | 0.21** | 0.02 | 0.01 |  |  |
| 15. Multimorbidity (0.00–16.00) | 2.7 (2.3) | 0.11** | 0.09** | 0.10** | –0.07** | –0.06** | 0.01 | –0.08** | 0.07** | 0.00 | –0.01 |  |  |
| 16. Religious Activity (1.00–5.00) | 2.7 (1.2) | –0.02 | –0.10** | –0.07** | 0.07** | –0.04* | 0.05** | 0.05** | –0.05* | 0.02 | 0.01 |  |  |
| 17. Religious at Home (1.00–5.00) | 4.6 (1.0) | 0.05** | 0.05* | 0.05** | 0.05** | 0.00 | –0.01 | –0.04* | 0.04* | –0.04* | 0.00 |  |  |
| 18. Religious Importance (1.00–4.00) | 3.9 (0.4) | 0.01 | 0.08** | 0.04* | 0.05** | –0.02 | 0.00 | –0.03 | 0.03 | 0.01 | –0.01 |  |  |

*Note.* **p* < .05, ***p* < .001. M = Mean; SD = Standard Deviation.

Intercorrelations (Part 2)

|  | Intercorrelations | | | | | | | | | | | | | | |  |
| --- | --- | --- | --- | --- | --- | --- | --- | --- | --- | --- | --- | --- | --- | --- | --- | --- |
| Variables (range) |  | 11 | 12 | 13 | 14 | 15 | 16 | 17 | 18 | | | | |  | |  |
| 12. Emotional Support (0.00–6.00) |  | 0.13** |  |  |  |  |  |  |  |  |  |  |  | |  | |
| 13. Instrumental Support (0.00–4.00) |  | –0.05** | 0.00 |  |  |  |  |  |  |  |  |  |  | |  | |
| 14. ADL limitations (0.00–24.00) |  | –0.28 | –0.04* | –0.12** |  |  |  |  |  |  |  |  |  | |  | |
| 15. Multimorbidity (0.00–16.00) |  | –0.03* | –0.01 | –0.07** | 0.20** |  |  |  |  |  |  |  |  | |  | |
| 16. Religious Activity (1.00–5.00) |  | 0.23* | 0.04* | 0.04* | –0.22** | 0.01 |  |  |  |  |  |  |  | |  | |
| 17. Religious at Home (1.00–5.00) |  | 0.17** | 0.08** | –0.07** | 0.01 | 0.07** | 0.11** |  |  |  |  |  |  | |  | |
| 18. Religious Importance (1.00–4.00) |  | 0.02 | 0.04* | –0.02 | 0.02 | 0.02 | 0.17** | 0.25** |  |  |  |  |  | |  | |

*Note*. **p* < .05, ***p* < .001. M = Mean; SD = Standard Deviation.

**Table S-3**

*Additional representative quotes not mentioned in the text.*

| **Categories** | **Representative Quotes** |
| --- | --- |
| Area of Residence | *“I don’t like to live in Yangon. (…) The [tennis] players are not very intimate with me, so I cannot make any friends if I lived there. If I were young, I could do it. But I’m old, and other people don’t want to play with old people.”* – Participant 2 |
| Financial Situation | *“If they [lonely people] needn’t worry about eating, wearing clothes, living, that would be better for them. Now, if they don’t have anyone to support them, they will feel down, nobody there to give them food.”* – Participant 3 |
| Family | *“When my family comes to me, I don’t feel lonely. I also never feel upset. I am happy. Very happy. I’m happy because I have people around me, my children.”* – Participant 7  *“My wife and I were separated for about 30 years. (...) She was moved to Naypyidaw, and we didn’t live together again. When I was young, that was difficult, but now due to old age I don’t want that anymore. When my wife comes, she comes, when she doesn’t come, that’s okay.”* – Participant 2 |
| Community | *“I am always involved in the community. (…) I don’t have time to feel lonely.”* – Participant 3 |
| Support | *“All of my children, they never leave me alone. They always come and ask me “how are you?” and always come and talk to me. They come to me when they need help, and when I’m sick, they come too. And they help me. I never feel loneliness.”* – Participant 7  *“Loneliness is when you don’t have relationships. (…) And when nobody cares for you, it makes you weak, or a few people.”–* Participant 3 |
| Health | *“If you stay alone, you will feel very dark, and your life will be very short.”* – Participant 2  *“When you are alone and lonely, you might feel very down. You can become sick from loneliness*.” – Participant 3  *“My health is not so good anymore, I cannot walk everywhere, my back hurts. And now my eyes are not good. Also, last week, I had pain in my jaw, I don’t know if it’s related to my teeth. But I don’t have money to buy medicine.”* – Participant 5 |
